# Roles of NO Signaling in Long-Term Memory Formation in Visual Learning in an Insect

**DOI:** 10.1371/journal.pone.0068538

**Published:** 2013-07-24

**Authors:** Yukihisa Matsumoto, Daisuke Hirashima, Kanta Terao, Makoto Mizunami

**Affiliations:** 1 Graduate School of Life Science, Hokkaido University, Sapporo, Japan; 2 Graduate School of Life Sciences, Tohoku University, Sendai, Japan; University of Houston, United States of America

## Abstract

Many insects exhibit excellent capability of visual learning, but the molecular and neural mechanisms are poorly understood. This is in contrast to accumulation of information on molecular and neural mechanisms of olfactory learning in insects. In olfactory learning in insects, it has been shown that cyclic AMP (cAMP) signaling critically participates in the formation of protein synthesis-dependent long-term memory (LTM) and, in some insects, nitric oxide (NO)-cyclic GMP (cGMP) signaling also plays roles in LTM formation. In this study, we examined the possible contribution of NO-cGMP signaling and cAMP signaling to LTM formation in visual pattern learning in crickets. Crickets that had been subjected to 8-trial conditioning to associate a visual pattern with water reward exhibited memory retention 1 day after conditioning, whereas those subjected to 4-trial conditioning exhibited 30-min memory retention but not 1-day retention. Injection of cycloheximide, a protein synthesis inhibitor, into the hemolymph prior to 8-trial conditioning blocked formation of 1-day memory, whereas it had no effect on 30-min memory formation, indicating that 1-day memory can be characterized as protein synthesis-dependent long-term memory (LTM). Injection of an inhibitor of the enzyme producing an NO or cAMP prior to 8-trial visual conditioning blocked LTM formation, whereas it had no effect on 30-min memory formation. Moreover, injection of an NO donor, cGMP analogue or cAMP analogue prior to 4-trial conditioning induced LTM. Induction of LTM by an NO donor was blocked by DDA, an inhibitor of adenylyl cyclase, an enzyme producing cAMP, but LTM induction by a cAMP analogue was not impaired by L-NAME, an inhibitor of NO synthase. The results indicate that cAMP signaling is downstream of NO signaling for visual LTM formation. We conclude that visual learning and olfactory learning share common biochemical cascades for LTM formation.

## Introduction

Insects are useful animals for the study of molecular and cellular mechanisms of learning [1–4]. Most previous studies on mechanisms of insect learning have focused on olfactory learning, and mechanisms of other forms of learning, such as visual learning, have remained largely unknown. Visual learning capability of insects was first demonstrated by von Frisch [5], who showed that honey bee foragers learn color and shape of profitable flowers and use the memory for revisiting them after returning to the hive. Subsequent studies on many insects, including honey bees [6,7], fruit-flies [8,9], butterflies [10] and crickets [11–13], demonstrated their capability to learn color, shape and other features of objects. Moreover, sophisticated forms of visual learning, such as visual landmark (spatial) learning [14–16], object categorization [17] and rule learning [6], have been demonstrated in some insects. Little is known, however, about the molecular and neural mechanisms of visual learning in insects except that the role of cAMP signaling in neurons of the central complex in formation of short-term visual memory has been suggested in the fruit-fly *Drosophila* [18–20]. This is in contrast to the accumulation of information on molecular and cellular mechanisms of olfactory learning in some species of insects [1–4,21].

In olfactory learning in insects, the mechanisms of formation of long-term memory (LTM) have been examined in detail [2]. LTM is defined as a protein synthesis-dependent phase of memory lasting from one day to a lifetime. It is usually formed by multiple pairing trials but not by a single trial. LTM storage is accomplished by enduring changes in synaptic strength that require transcription and translation of genes [22]. In insects, as in mollusks [22], this is achieved by activation of cAMP signaling and resulting phosphorylation of the transcription factor cAMP responsible element-binding protein (CREB), which lead to translation of genes that are necessary for modification of synaptic transmission [22,23].

The roles of the cAMP pathway in LTM formation are often supplemented by other signaling pathways such as the nitric oxide (NO)-cGMP pathway in invertebrates [24,25], as in vertebrates [26,27]. In insects, this was demonstrated in olfactory learning in crickets [28,29], honey bees [30,31] and cockroaches [32] and in tactile learning in honey bees [33]. NO is a membrane-permeable intercellular signaling molecule produced by NO synthase (NOS) [34]. NO diffuses into neighboring neurons and stimulates soluble guanylyl cyclase, an enzyme producing cGMP. In crickets, we have provided pharmacological evidence suggesting that cAMP signaling is a downstream target of NO-cGMP signaling, namely, the NO-cGMP signaling stimulates adenylyl cyclase, via activation of cyclic nucleotide-gated (CNG) channel and calcium-calmodulin signaling, for LTM formation [28,29]. We confirmed the role of NO in olfactory LTM formation in a study using the RNA interference (RNAi) technique: crickets with a reduced level of *NOS* mRNA expression in the brain by RNAi exhibited impairment of olfactory LTM formation [35].

In this study, we examined possible participation of NO-cGMP signaling and cAMP signaling in LTM formation in visual pattern learning in crickets and investigated whether the finding in olfactory learning that NO signaling stimulates cAMP signaling for LTM formation is applicable to visual learning. The results of this study suggest that the biochemical cascade underlying visual LTM formation is fundamentally similar to that of olfactory LTM formation, providing a solid basis for further elucidating molecular mechanisms of visual learning in insects.

## Materials and Methods

### Insects

Adult male crickets, *Gryllus* *bimaculatus* , at 1-2 weeks after the imaginal molt were used. They were reared in a 12 h: 12 h light: dark cycle (photophase: 8:00-20:00) at 27±2C and were fed a diet of insect pellets and water *ad libitum*. Four days before the start of the experiment, a group of 20-30 animals was placed in a container and fed a diet of insect pellets *ad libitum* but deprived of drinking water to enhance their motivation to search for water. On the day of the experiment, they were individually placed in 100-ml glass beakers.

### Conditioning

**Figure 1:**
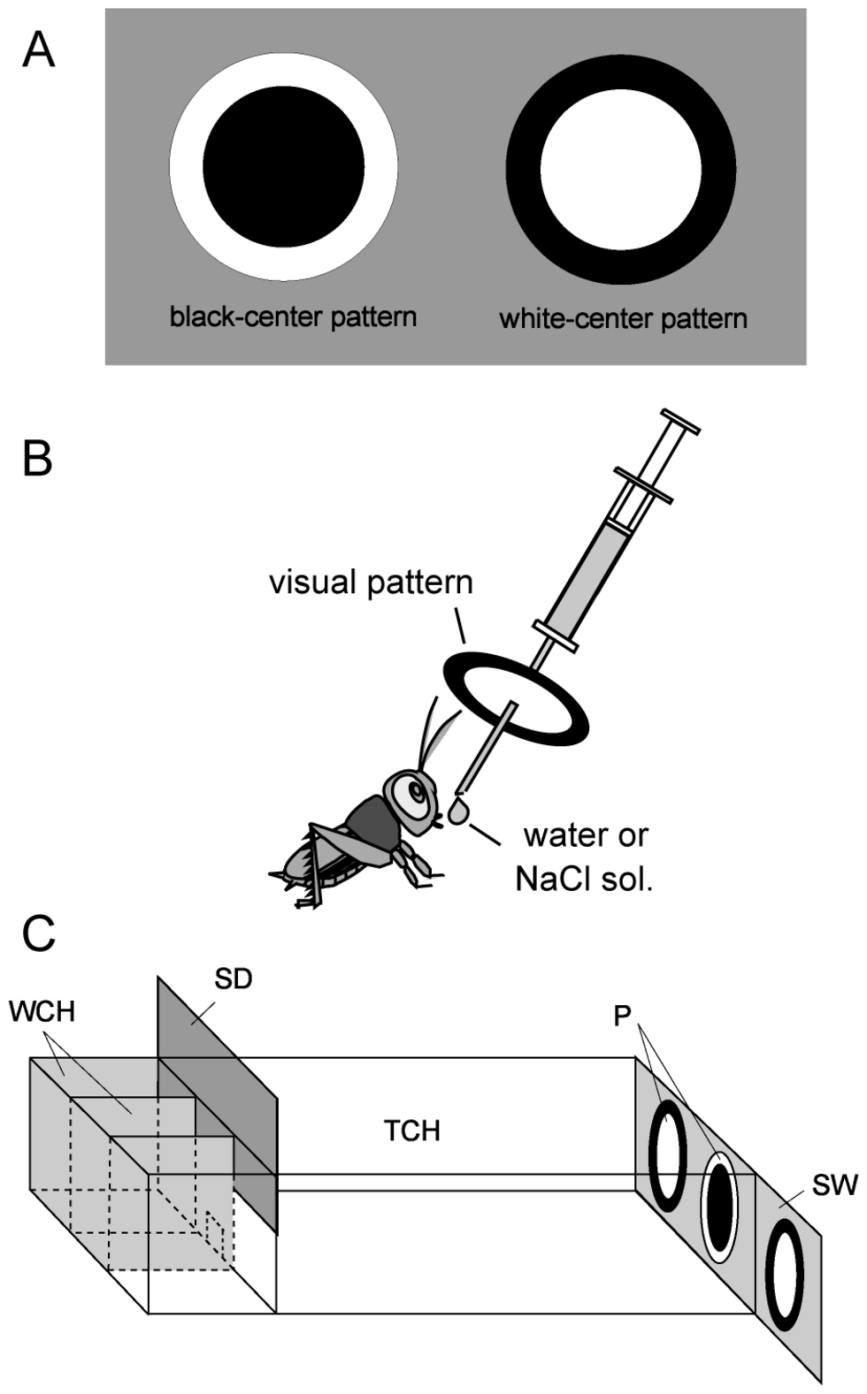
Experimental procedure. (A) Black-center and white-surround pattern (black-center pattern, left) and white-center and black-surround pattern (white-center pattern, right) used as conditioning stimuli. (B) Procedure for conditioning. Crickets were individually placed in a beaker (not shown). A syringe containing water or sodium chloride solution was used for conditioning. A white-center or black-center pattern was attached to the needle of the syringe. The pattern was placed in front of the cricket’s head and then a drop of water or sodium chloride solution was given to the mouth. (C) Apparatus used for the preference test. WCH, waiting chambers; TCH, test chamber; SD, sliding door; P, visual pattern; SW, sliding wall.

We used a classical conditioning and operant testing procedure for visual pattern conditioning [11]. In short, a black-center and white-surround pattern (black-center pattern) or a white-center and black-surround pattern (white-center pattern) (Figure 1A) was used as a conditioned stimulus (CS) and water or 20% sodium chloride solution was used as an appetitive or aversive unconditioned stimulus (US). A syringe containing water or sodium chloride solution was used for appetitive or aversive conditioning (Figure 1B). A pattern was attached to the needle of the syringe at 1 cm from the tip. The pattern was presented above the cricket’s head and then water reward or sodium chloride punishment was presented to the mouth for appetitive or aversive conditioning, respectively. The crickets were subjected to 4 or 8 pairing trials for appetitive conditioning or 12 pairing trials for aversive conditioning. The inter-trial interval (ITI) was 5 min.

### Preference test

The procedure for the dual-choice visual pattern preference test was described previously [11]. In short, all groups of animals were subjected to preference tests before and after conditioning. Two white-center patterns and one black-center pattern were presented on a grey sliding wall at the end of the test chamber, and two of the three patterns could be presented at the same time (Figure 1C). A cricket was transferred to the waiting chamber and left for 4 min. Then the cricket was allowed to enter the test chamber and the test started. Two min later, the relative positions of the black-center and white-center patterns were changed by sliding the wall. The test lasted for 4 min. If the total visiting time was less than 10 sec, we considered that the animal was less motivated to visit patterns and the data were rejected. We observed no significantly different levels of conditioning effect between the group in which a black-center pattern was used as CS and the group in which a white-center pattern was used as CS, and thus the data from the two groups were pooled.

### Pharmacology

Animals were each injected with 3 µl of saline containing drugs into the hemolymph of the head using a microsyringe. Nw-nitro-L-arginine methyl ester (L-NAME), Nw-nitro-D-arginine methyl ester (D-NAME), 8-bromoguanosine 3': 5'-cyclic monophosphate (8-br-cGMP), 8-bromoadenosine 3',5'-cyclic monophosphate (8-br-cAMP), cycloheximide (CHX), dimethyl sulphoxide (DMSO) and 2',5'-dideoxyadenosine (DDA) were purchased from SIGMA (Tokyo, Japan), and (±)-N-[(E)-4-Ethyl-2-[(Z)-hydroxyimino] -5-nitro-3-hexene-1-yl]-3-pyridinecarboxamide (NOR4) was purchased from Wako (Tokyo, Japan). NOR4 was dissolved in cricket saline [36] containing 0.1% DMSO, and all other drugs were dissolved in cricket saline.

### Data analysis

A pattern was considered to have been visited when the cricket probed the pattern with its mouth or pulpi. The time spent visiting each pattern was measured cumulatively. In appetitive conditioning, relative preference of each animal was determined using the preference index (PI) for rewarded pattern, defined as t_r_/(t_r_+t_nr_)x100(%) for visual pattern conditioning, where t_r_ was the time spent exploring the pattern associated with reward and t_nr_ was the time spent exploring the pattern not associated with reward. In aversive conditioning, relative preference was determined using the PI for unpunished pattern, defined as t_np_/(t_np_+t_p_)x100, where t_np_ was the time spent exploring the pattern not associated with punishment and t_p_ was the time spent exploring the pattern associated with punishment [11]. We compared visual pattern preferences after training with those before training in each animal group by Wilcoxon signed-rank test (WCX test). We found no significant differences in visual pattern preferences among the different groups of animals before training (Kruskal-Wallis test, p>0.5).

## Results

### Formation of protein synthesis-dependent LTM in visual learning

**Figure 2:**
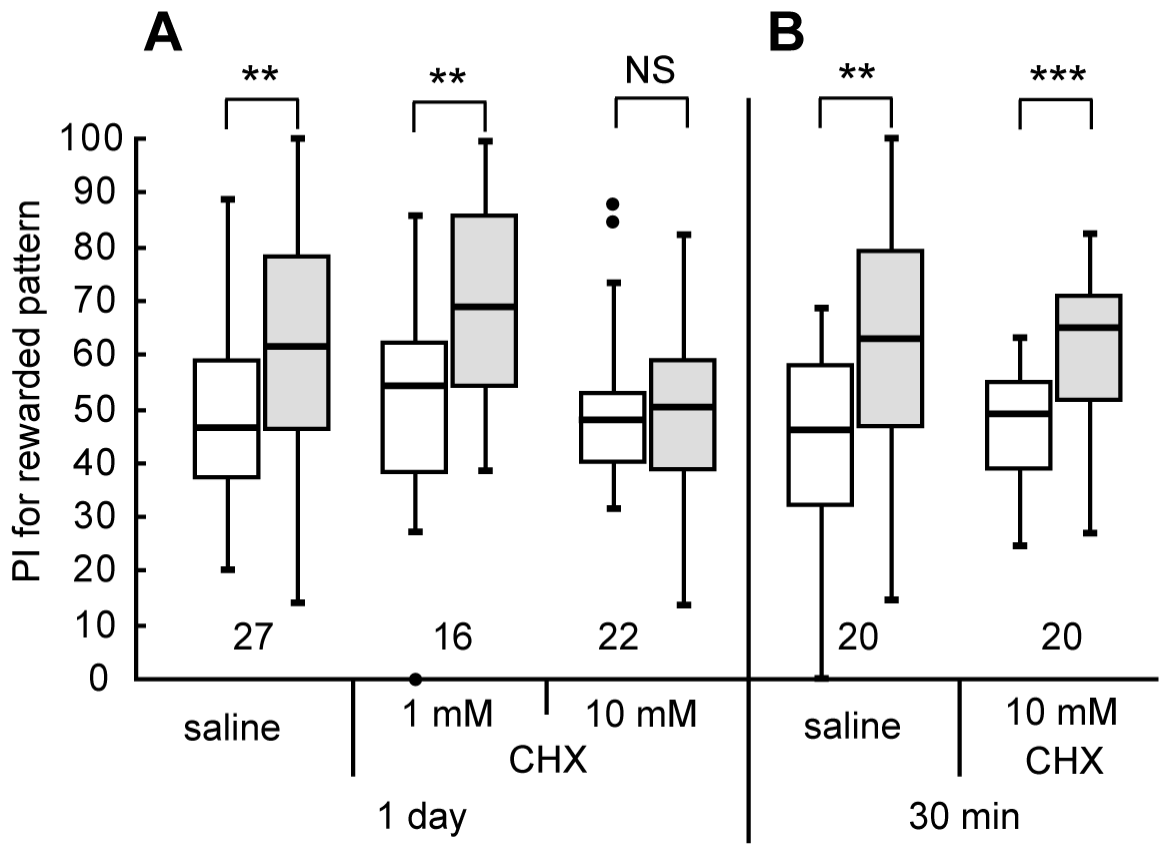
Effects of cycloheximide on formation of 1-day memory after visual pattern conditioning. (A) Three groups of animals were each subjected to injection of 3 μl of saline or saline containing 1 mM or 10 mM cycloheximide (CHX) at 20 min prior to 8-trial conditioning in which a visual pattern was paired with water reward. Relative preference between the rewarded pattern and control pattern was tested before and at 1 day after training. (B) Another two groups were each injected with 3 μl of saline or saline containing 10 mM CHX at 20 min prior to 8-trial visual conditioning. Relative preference between the rewarded pattern and control pattern was tested before and at 30 min after training. Preferences indexes (PIs) for the rewarded patterns before (white bars) and after (grey bars) training are shown as box and whisker diagrams and are statistically compared. The line in the box is the median and the box represents the 25-75 percentiles. Whiskers extend to extreme values as long as they are within a range of 1.5× box length from the upper or lower quartiles. Any data not included between the whiskers are plotted as outliers with dots. The results of statistical comparisons of visual pattern preferences before and after training are shown by asterisks (*** P<0.001, ** P<0.01, NS P>0.05, WCX test). The number of animals tested is shown at each data point in this figure and in subsequent figures.

First, we studied whether the formation of 1-day memory after visual conditioning can be characterized as protein synthesis-dependent LTM. We used 8-trial conditioning to associate a visual pattern with water reward, with an inter-trial interval of 5 min, because we observed that it leads to formation of 1-day memory, whereas 4-trial conditioning leads to formation of 30-min memory but not 1-day memory [11]. Three groups of animals were each injected with 3 µl of cricket saline or saline containing 1 mM or 10 mM cycloheximide, a protein synthesis inhibitor, into the hemolymph of the head at 20 min prior to 8-trial conditioning. The relative preference between the conditioned pattern and control pattern was tested before and 1 day after conditioning. The group injected with saline exhibited significantly increased preference for the conditioned pattern at 1 day after conditioning compared to that before conditioning (Figure 2A; W=72, p=0.0039, WCX test, the sample number shown in legends). On the other hand, the group injected with 10 mM cycloheximide exhibited no significant level of 1-day memory retention (W=117, p=0.77, WCX test). The group injected with 1 mM cycloheximide exhibited a significant level of 1-day retention (W=9, p=0.0010, WCX test), indicating that the effect of cycloheximide is dose-dependent. We conclude that memory at 1 day after 8-trial visual conditioning can be characterized as protein synthesis-dependent LTM.

To determine whether the effect of cycloheximide is specific to LTM formation, another two groups of animals were injected with 3 μl of saline or saline containing 10 mM cycloheximide at 20 min prior to 8-trial conditioning, and their preferences were tested before and 30 min after conditioning. The cycloheximide-injected group exhibited a significant level of 30-min memory retention (Figure 2B, W=20, p=0.00071, WCX test), as did the saline-injected group (W=30, p=0.0037, WCX test), indicating that protein synthesis is not required for 30-min memory formation. Animals injected with cycloheximide, or any other drugs used in this study, exhibited normal responses to appetitive stimuli during training: They drank water eagerly when water was presented to the mouth, as did intact or saline-injected crickets. Drug-injected crickets also exhibited normal locomotory activity and exploration of odor sources during testing. The results indicate that cycloheximide did not impair 1) sensory and motor functions necessary for normal learning performance, 2) initial acquisition of memory or 3) memory retention up to 30 min after conditioning. These observations are in accordance with findings in olfactory learning [35,36].

### Participation of NO-cGMP signaling in visual LTM formation

**Figure 3:**
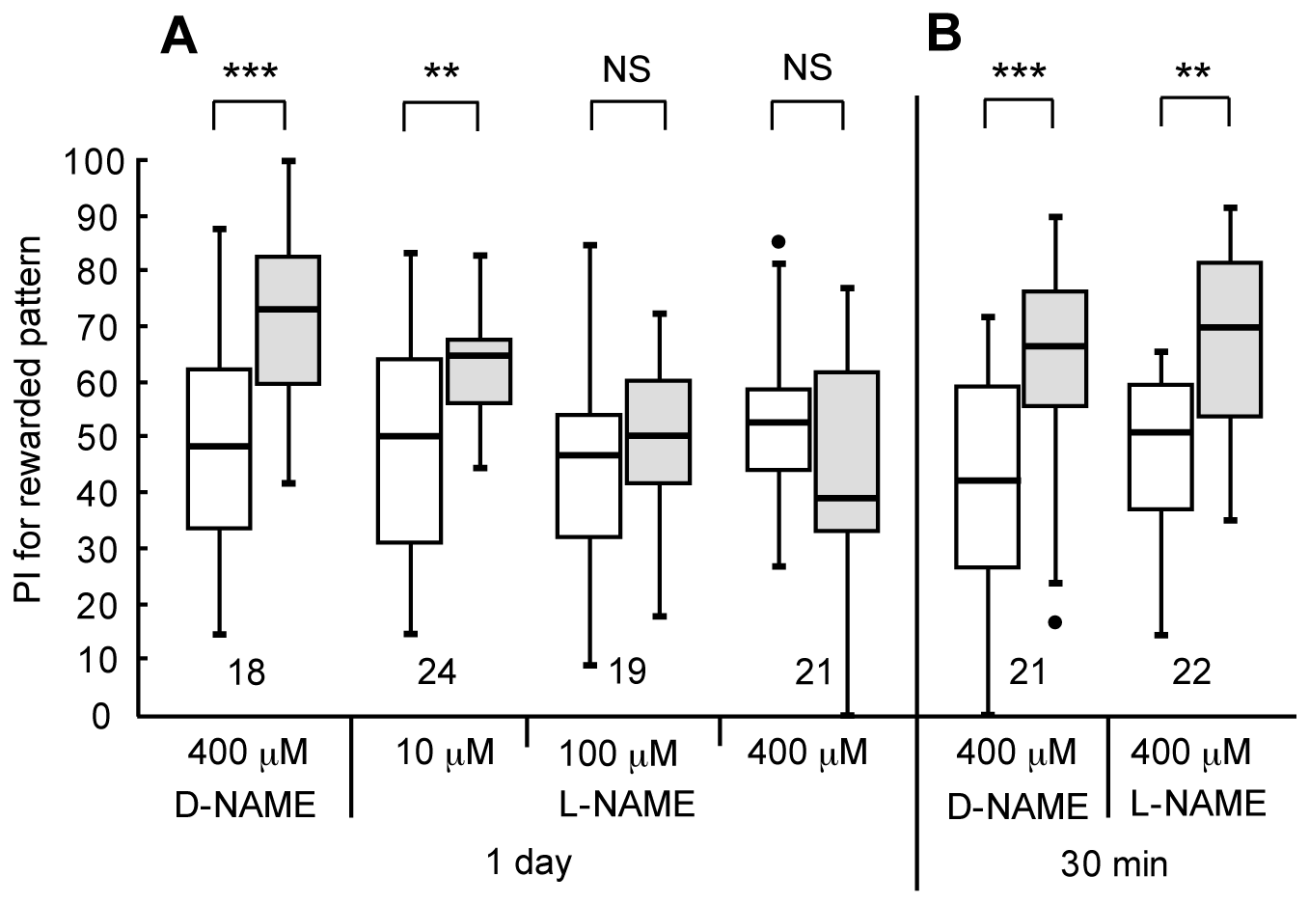
Effects of L-NAME on visual LTM formation. (A) Four groups of animals were each injected with 3 μl of saline containing 400 μM D-NAME or 20 μM, 100 μM or 400 μM L-NAME at 20 min prior to 8-trial visual conditioning. Relative preference between the rewarded pattern and control pattern was tested before and at 1 day after training. (B) Another two groups were each injected with 3 μl of saline containing 400 μM D-NAME or 400 μM L-NAME at 20 min prior to conditioning. Relative preference between the rewarded pattern and control pattern was tested before and at 30 min after conditioning. PIs for the rewarded pattern before (white bars) and after (grey bars) training are shown as box plots, and the results of statistical comparisons between them are indicated (*** P<0.001, ** P<0.01, NS P>0.05, WCX test).

In order to determine possible roles of NO-cGMP signaling in visual LTM formation, we first studied the effect of L-NAME, a competitive inhibitor of NO synthase (NOS) [30]. Four groups of animals were each injected with 3 μl of saline containing 10 μM, 100 μM or 400 μM L-NAME or 400 μM D-NAME, an inactive isomer, into the hemolymph at 20 min prior to 8-trial conditioning, and their preferences were tested before and 1 day after conditioning. The D-NAME-injected group (Figure 3A, W=12, p=0.00053, WCX test) and the 10 μM L-NAME-injected group (W=59, p=0.0079, WCX test) exhibited significant levels of 1-day retention. However, the group injected with 100 μM (W=60, p=0.17, WCX test) or 400 μM L-NAME (W=156, p=0.17, WCX test) did not, indicating that NO synthesis is necessary for LTM formation. Another two groups were each injected with 3 μl of saline containing 400 μM L-NAME or 400 μM D-NAME at 20 min prior to 8-trial-trial conditioning and their preferences were tested before and 30-min after conditioning. The L-NAME-injected group exhibited a significant level of 30-min retention (Figure 3B, W=21, p=0.0011, WCX test), as did the D-NAME-injected group (W=14, p=0.00010, WCX test), indicating that NO synthesis is not needed for 30-min memory formation. The dose of L-NAME necessary for achieving blockade of LTM formation was 3 μl x 100 μM, which approximately corresponds to a concentration of 0.4 μM after diffusion, calculated from the approximate body weight of 850 mg. This concentration is comparable to that used to study the effect of L-NAME on LTM in olfactory learning in crickets (1.5 μM [28]) and honey bees (1.0 μM [30]).

**Figure 4:**
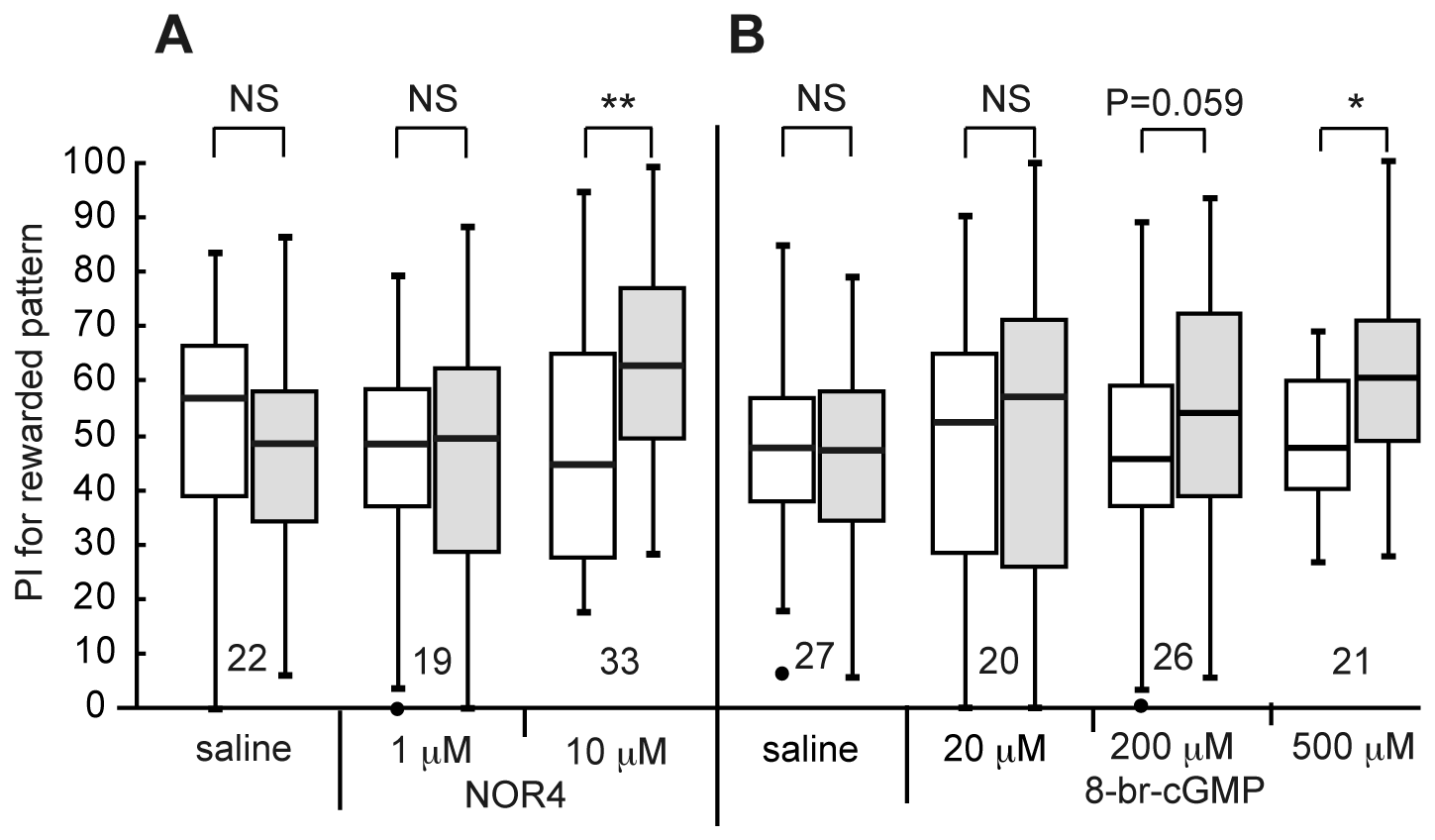
Effects of NOR4 and 8-br-cGMP on visual LTM formation. (A) At 20 min prior to 4-trial visual conditioning, three groups of animals were each injected with 3 μl of saline (containing 0.1% DMSO) or saline containing 1 μM or 10 μM NOR4 (and 0.1% DMSO). (B) At 20 min prior to 4-trial conditioning, another four groups were each injected with 3 µl of saline or saline containing 20 μM, 200 μM or 500 μM 8-br-cGMP. Relative preference between the rewarded pattern and control pattern was tested before and at 1 day after conditioning. PIs for the rewarded pattern before (white bars) and after (grey bars) training are shown as box plots, and the results of statistical comparison between them are indicated (** p<0.01, * p<0.05, NS p>0.05, WCX test).

Next, we tested whether injection of NOR4, an NO donor, prior to 4-trial conditioning could facilitate LTM formation. Three groups of animals were each injected with 3 μl of saline (containing 0.1% DMSO) or saline containing 1 μM or 10 μM NOR4 (and 0.1% DMSO) prior to 4-trial conditioning, and their preferences were tested before and 1 day after conditioning. The groups injected with 3 μ1 of saline (Figure 4A, W=135, p=0.80, WCX test) and saline containing 1 μM NOR4 (W=88, p=0.93, WCX test) prior to 4-trial conditioning exhibited no significant level of 1-day retention. On the other hand, the group injected with 3 µ1 of saline containing 10 µM NOR4 prior to 4-trial conditioning exhibited significant 1-day retention (W=115, p=0.0024, WCX test), indicating that externally applied NO facilitates LTM formation.

In olfactory conditioning in crickets, we concluded that the effect of an NO donor for inducing LTM is mediated via stimulation of soluble guanylyl cyclase and resulting production of cGMP [28], and we next studied whether injection of 8-br-cGMP, a membrane-permeable cGMP analogue, could stimulate visual LTM formation. Three groups of animals were each injected with 3 μ1 of saline or saline containing 20 μM, 200 μM or 500 μM 8-br-cGMP prior to 4-trial conditioning, and their preferences were tested before and 1 day after conditioning. The groups injected with saline (Figure 4B, W=216, p=0.98, WCX test) and saline containing 20 μM 8-br-cGMP (W=100, p=0.87, WCX test) exhibited no significant level of 1-day retention, and the level of 1-day retention of 200 μM 8-br-cGMP group was marginal (W=101, p=0.059, WCX test). On the other hand, 500 μM 8-br-cGMP group exhibited a significant level of 1-day retention (W=46, p=0.014, WCX test), indicating that externally applied cGMP facilitates LTM formation.

### Participation of cAMP signaling in visual LTM formation

**Figure 5:**
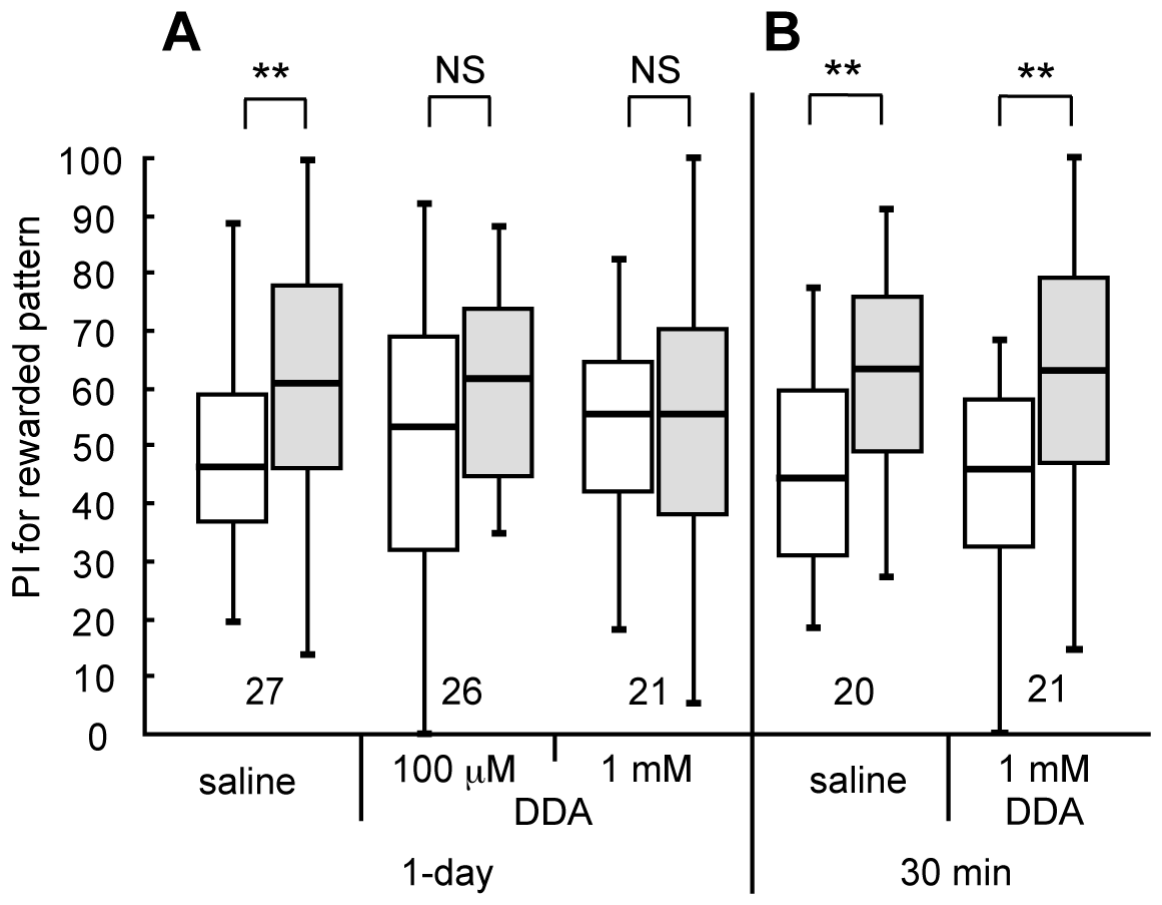
Effects of the adenylyl cyclase inhibitor DDA on visual LTM formation. (A) At 20 min prior to 8-trial visual conditioning, three groups of animals were each injected with 3 μl of saline or saline containing 100 μM or 1 mM DDA. Relative preference between the rewarded pattern and control pattern was tested before and at 1 day after conditioning. (B) At 20 min prior to 8-trial conditioning, another two groups were each injected with 3 μl of saline or saline containing 1 mM DDA. Relative preference between the rewarded pattern and control pattern was tested before and at 30 min after training. PIs for rewarded pattern before (white bars) and after (grey bars) training are shown as box plots, and the results of statistical comparison between them are indicated (** p<0.01; NS p>0.05, WCX test).

In olfactory conditioning in crickets, we showed that injection of DDA, an inhibitor of adenylyl cyclase [37], impairs LTM formation [28,29], and we investigated the effect of DDA on visual LTM formation in this study. Three groups of animals were each injected with 3 μl of saline or saline containing 100 μM or 1 mM DDA prior to 8-trial conditioning, and their preferences were tested before and 1 day after conditioning. The groups injected with saline (Figure 5A, W=72, p=0.0039, WCX test) exhibited significant levels of 1-day retention. In contrast, the groups injected with 100 μM DDA (W=113.5, p=0.12, WCX test) and 1 mM DDA (W=104, p=1.0, WCX test) did not exhibit significant levels of 1-day retention, indicating that cAMP production participates in LTM formation (Figure 5A). Another two groups of crickets were each injected with 3 μl of saline or saline containing 1 mM DDA prior to 8-trial conditioning, and their preferences were tested before and 30 min after conditioning. The DDA-injected group exhibited a significant level of 30-min retention (Figure 5B, W=31, p=0.0022, WCX test), as did the saline-injected group (W=30, p=0.0037, WCX test), indicating that cAMP does not participate in 30-min memory formation.

**Figure 6:**
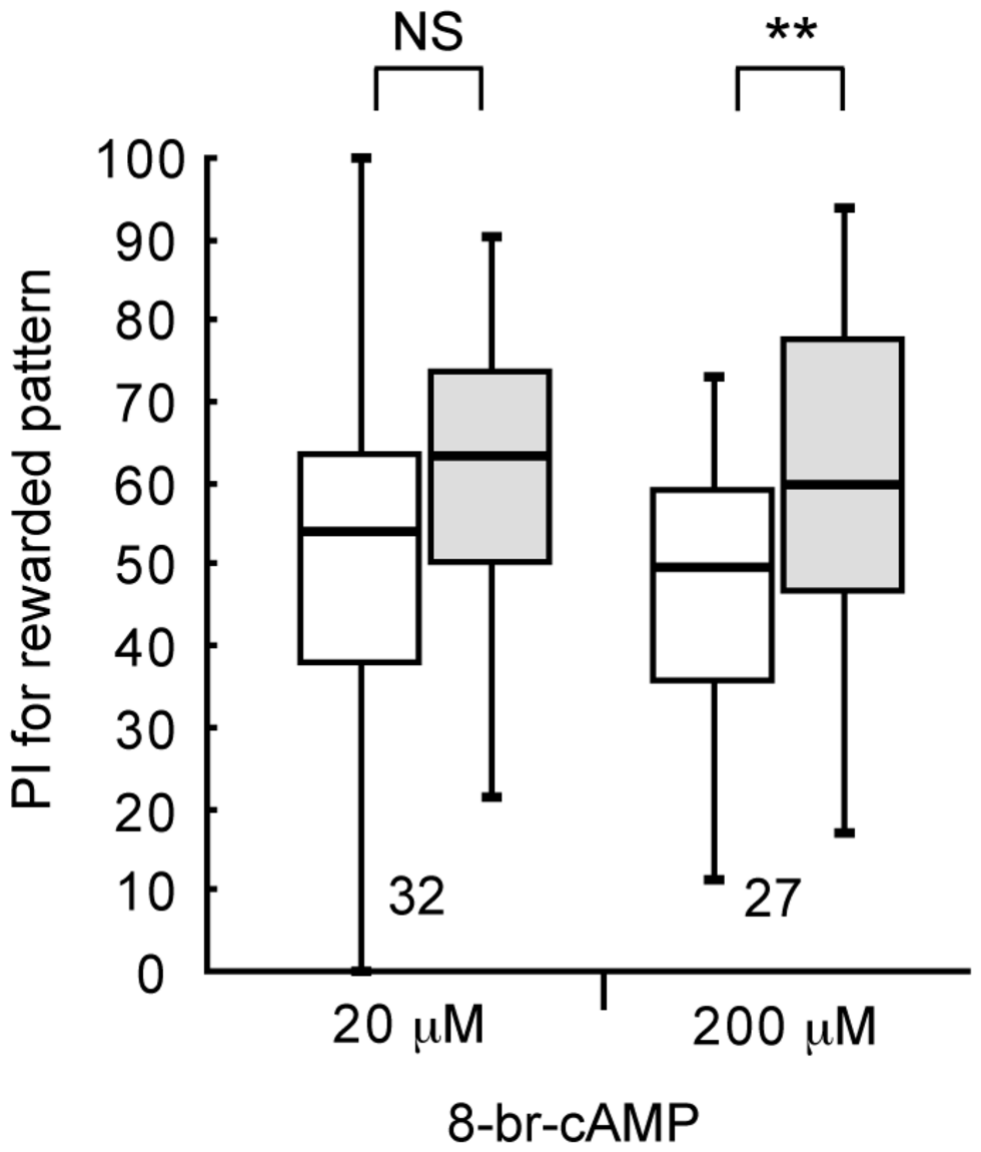
Effects of 8-br-cAMP on visual LTM formation. At 20 min prior to 4-trial visual conditioning, two groups of animals were each injected with 3 μl of saline or saline containing 20 μl or 200 μl 8-br-cAMP. Relative preference between the rewarded pattern and control pattern was tested before and at 1 day after conditioning. PIs for rewarded pattern before (white bars) and after (grey bars) training are shown as box plots, and the results of statistical comparison between them are indicated (** p<0.01, NS p>0.05, WCX test).

We next tested whether injection of 8-br-cAMP, a membrane-permeable cAMP analogue, can facilitate visual LTM formation. Two groups of animals were each injected with 3 μl or saline containing 20 μM or 200 μM 8-br-cAMP at 20 min prior to 4-trial conditioning, and their preferences were tested before and 1 day after conditioning. The group injected with 20 μM 8-br-cAMP exhibited no significant level of 1-day retention (Figure 6, W=206, p=0.29, WCX test), as in the case of the saline-injected group (see Figure 4). In contrast, the 200 μM 8-br-cAMP-injected group exhibited a significant level of 1-day retention, (W=72, p=0.0039, WCX test), indicating that externally applied cAMP facilitates LTM formation.

### Relationship between NO signaling and the cAMP signaling for visual LTM formation

**Figure 7:**
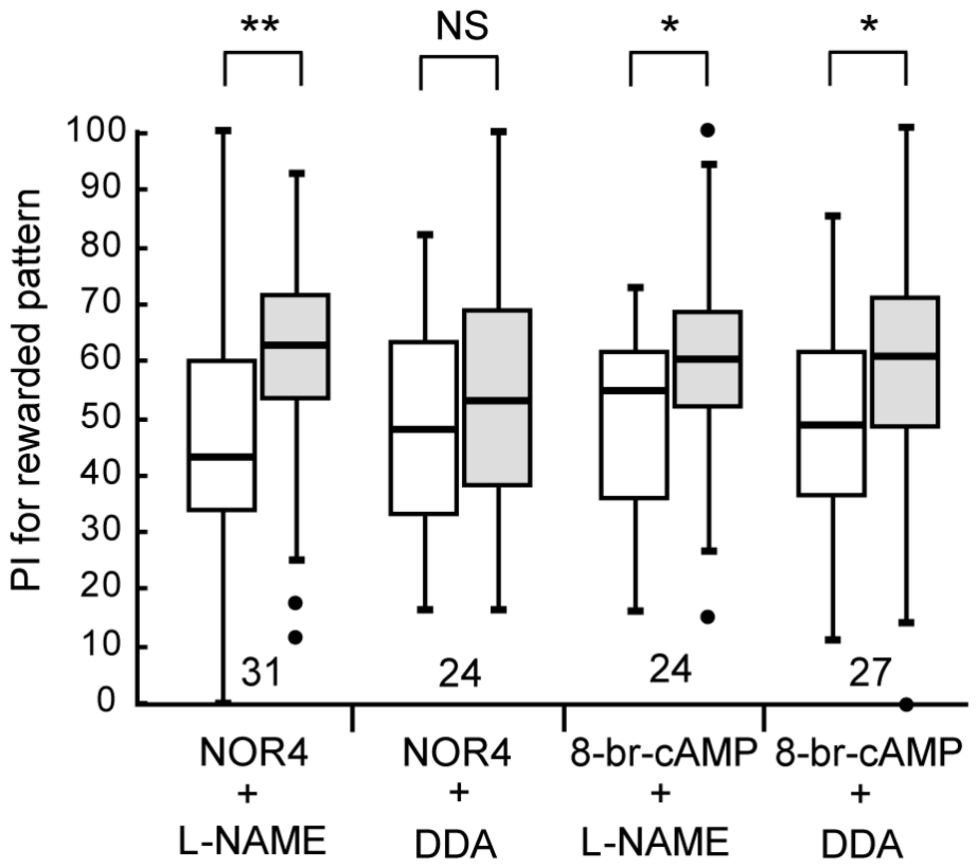
Effects of co-injection of LTM-inducing drugs and LTM-inhibiting drugs on visual LTM induction. At 20 min prior to 4-trial visual conditioning, four groups of animals were each co-injected with 3 μl of saline containing NOR4 (10 μM) and L-NAME (400 μM), NOR4 (10 μM) and DDA (1 mM), 8-br-cAMP (200 μM) and L-NAME (400 μM), or 8-br-cAMP (200 µM) and DDA (1 mM). Relative preference between the rewarded pattern and control pattern was tested before and at 1 day after conditioning. The PIs for the rewarded pattern before (white bars) and after (grey bars) training are shown as box plots, and the results of statistical comparison are indicated (** p<0.01, * p<0.05, NS p>0.05, WCX test).

We next studied the relationship between NO signaling and cAMP signaling for visual LTM formation by co-injecting their activators and inhibitors. Four groups of crickets were each injected with 3 μ1 of saline containing (1) NOR4 (10 μM) and L-NAME (400 μM), (2) NOR4 (10 μM) and DDA (1 mM), (3) 8-br-cAMP (200 μM) and L-NAME (400 μM) or (4) 8-br-cAMP (200 μM) and DDA (1 mM) prior to 4-trial conditioning, and their preferences were tested before and 1 day after conditioning. The group injected with NOR4 and L-NAME exhibited a significant level of 1-day retention (Figure 7, W=91.5, p=0.0022, WCX test). This is not surprising since the effect of an NO donor for inducing LTM should not be blocked by inhibition of NOS. The group co-injected with NOR4 and DDA exhibited no significant level of 1-day retention (W=134, p=0.66, WCX test), suggesting that LTM induction by NO is mediated by cAMP production. The group co-injected with 8-br-cAMP and L-NAME exhibited a significant level of 1-day retention (W=72, p=0.025, WCX test), indicating that LTM induction by 8-br-AMP is not impaired by inhibition of NOS, in agreement with the suggestion that cAMP is downstream of NO for LTM formation. Finally, the group injected with 8-br-cAMP and DDA exhibited a significant level of 1-day retention (W=91, p=0.017, WCX test). This is again not surprising since the effect of 8-br-cAMP for inducing LTM should not be impaired by blockade of cAMP synthesis. We thus suggest that NO signaling stimulates cAMP production for LTM formation.

### Participation of NO in formation of aversive LTM

**Figure 8:**
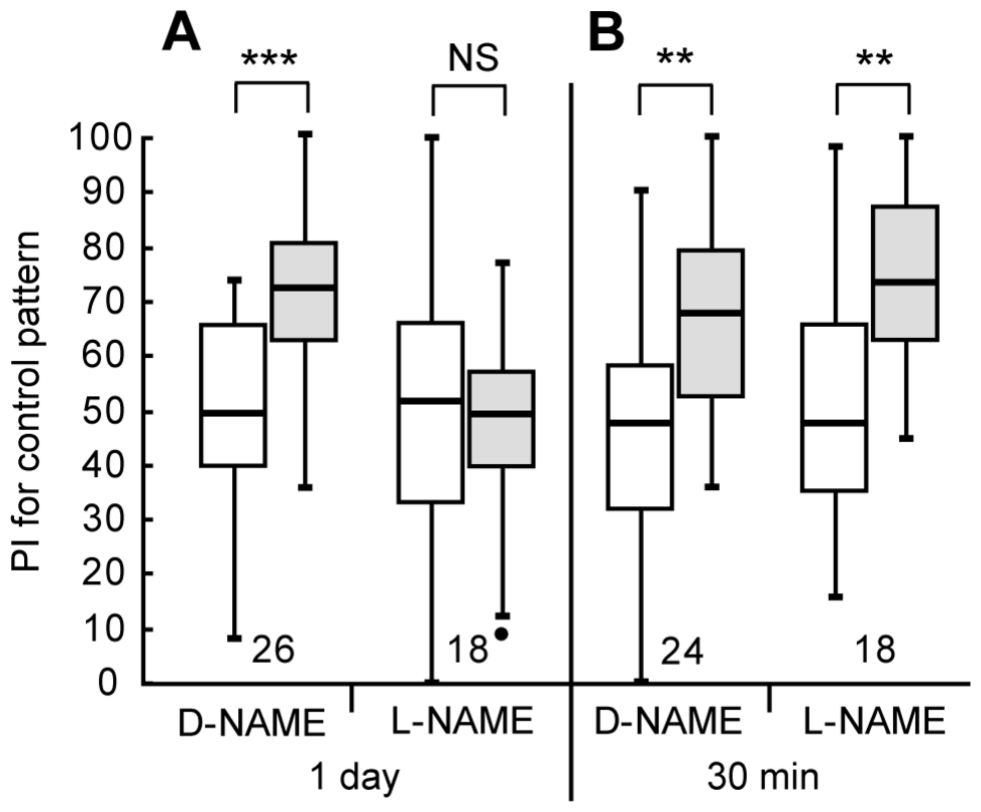
Effects of L-NAME on LTM formation in aversive visual conditioning. (A) At 20 min prior to 12-trial aversive conditioning to associate a visual pattern with sodium chloride punishment, two groups of animals were each injected with 3 μl of saline containing D-NAME (400 μM) or L-NAME (400 μM). Relative preference between the rewarded pattern and control pattern was tested before and at 1 day after training. (B) At 20 min prior to 12-trial conditioning, another two groups were each injected with 3 μl of saline containing D-NAME (400 μM) or L-NAME (400 μM). Relative pattern preference was tested before and at 30 min after training. The PIs for the control pattern before (white bars) and after (grey bars) training are shown as box plots: Notice that increased preference for the control pattern after training indicates decreased preference for the punished pattern. The results of statistical comparison are indicated (*** P<0.001; ** P<0.01; NS P>0.05, WCX test).

Finally, we studied whether NO participates in LTM formation in aversive visual learning. We previously observed that 12-trial conditioning to associate a visual pattern with sodium chloride punishment, with an inter-trial interval of 5 min, is necessary to achieve 1-day memory retention [11]. Two groups of animals were each injected with 3 μl of saline containing 400 μM L-NAME or 400 μM D-NAME at 20 min prior to 12-trial conditioning, and the preferences were tested before and 1 day after conditioning. The group injected with 400 μM D-NAME exhibited a significant level of 1-day retention (Figure 8A, W=40, p=0.00025, WCX test), but the group injected with 400 μM L-NAME exhibited no significant level of 1-day retention (W=91, p=0.51, WCX test; notice that the increased preference for the control pattern shown in Figure 8 indicates decreased preference for the conditioned pattern.). The results indicate that NO participates in formation of aversive visual LTM. Another two groups were each injected with 3 μl saline containing 400 μM L-NAME or 400 μM D-NAME at 20 min prior to 12-trial conditioning, and the preferences were tested before and at 30 min after conditioning. The L-NAME-injected group exhibited a significant level of 30-min retention (Figure 8B, W=15, p=0.0010, WCX test), as did the D-NAME-injected group (W=46, p=0.0020, WCX test), indicating that NO does not participate in 30-min aversive memory formation. We thus conclude that NO signaling participates in LTM formation in aversive visual learning.

## Discussion

Many species of insects exhibit visual learning for a variety of life activities, including feeding, predator avoidance and sexual behavior [6,38], but the molecular and neural mechanisms remain largely unknown. The aim of this study was to clarify signaling cascades underlying LTM formation in visual pattern learning in crickets. First, we found that 1-day memory after 8-trial visual conditioning is sensitive to a protein synthesis inhibitor, and we thus concluded that 1-day visual memory can be characterized as protein synthesis-dependent LTM. We next showed that blockade of production of NO or cAMP prior to 8-trial conditioning impairs LTM formation, whereas it does not impair formation of 30-min memory. We also found that animals subjected to 4-trial conditioning do not exhibit 1-day memory but that animals injected with an NO donor, cGMP analogue or cAMP analogue prior to 4-trial conditioning exhibit 1-day memory. We thus concluded that NO-cGMP signaling and cAMP signaling participate in visual LTM formation in crickets. The doses of L-NAME, 8-br-cGMP and 8-br-cAMP for inhibiting or inducing visual LTM were comparable to those for inhibiting or inducing olfactory LTM [28].

In olfactory learning in crickets, we have suggested that the NO-cGMP system stimulates the cAMP system, via activation of the CNG channel and calcium-calmodulin system, for LTM formation [28,29]. In one study, for example, we obtained evidence suggesting that the NO-cGMP system and cAMP system are arranged in series, not in parallel, for LTM formation [29]. In this study, we observed that induction of LTM by an NO donor is impaired by blockade of cAMP production in visual learning, in agreement with our observations in olfactory learning. We thus suggest that cAMP signaling is a downstream target of NO signaling in visual learning, as in olfactory learning, although further study is needed to convincingly demonstrate this. In conclusion, we suggest that visual learning and olfactory learning share common signaling cascades for LTM formation. To our knowledge, this study is the first study to show biochemical cascades underlying visual LTM formation in any insect, and it paves the way for further elucidation of the molecular and cellular mechanisms of visual learning.

We showed that NO signaling participates in the formation of both appetitive and aversive visual LTM. In our previous study on the roles of NO signaling in olfactory LTM formation, we used an elemental appetitive conditioning procedure [29] and a differential conditioning procedure to associate an odor with water reward and another odor with sodium chloride punishment [28,29] and, hence, it was unknown whether NO participates in LTM formation in aversive olfactory learning. Appetitive visual learning and aversive visual learning share common molecular mechanisms for LTM formation, although they differ in underling neurotransmitter mechanisms: we have shown that octopaminergic neurons participate in the former, whereas dopaminergic neurons participate in the latter [11,29].

It seems that the requirement of NO signaling for LTM formation differs in different insects. Critical roles of NO signaling in olfactory LTM formation have been found in crickets, honey bees [30,31] and cockroaches [32] but not in the fruit-fly *Drosophila* , on account of accumulation of information on molecular mechanisms of LTM formation in this species [1,2,23]. Moreover, it seems that the manner by which NO signaling participates in LTM formation differs in different insects. In honey bees, it has been proposed that cGMP and cAMP converge on protein kinase A (PKA), namely, the NO-cGMP system and cAMP system act synergistically and in parallel for olfactory LTM formation [31,39]. In crickets, however, the results of our pharmacological study were inconsistent with the proposal and strongly suggested serial arrangement of the NO-cGMP system and cAMP system [29]. The diversity of molecular mechanisms of LTM formation among different insects, and its possible functional and evolutionary significance, should be the subject of our future research.

It also appears that there is diversity in the signaling cascade underlying STM formation among insects. In crickets, we obtained evidence to suggest that cAMP signaling does not participate in STM formation in visual pattern learning (this study) and olfactory learning [6,8]. In the fruit-fly *Drosophila* , on the other hand, participation of cGMP-PKG (cGMP-dependent protein kinase) signaling in aversive STM formation in visual pattern learning has been proposed [19] and, moreover, the role of cAMP signaling in STM formation has been well documented. For the latter, for example, *ruta* *baga* mutants defective in adenylyl cyclase exhibit a memory decay within a few minutes after conditioning in olfactory learning [2] and visual pattern learning [18,20]. Comparative studies on molecular mechanisms for STM formation among insects should also emerge as an interesting research subject.

Elucidation of brain areas participating in visual LTM formation is one of major subjects of our study. The neural mechanisms of visual learning are poorly understood in insects, except that it has been suggested that cAMP signaling in neurons of the central complex participates in STM formation in visual pattern learning in the fruit-fly *Drosophila* [18,20]. In crickets, however, whether the central complex participates in visual pattern learning is an open question because we obtained evidence to suggest that cAMP signaling does not participate in visual STM formation. The finding in the present study that NO signaling participates in visual LTM formation should provide a starting point for elucidating its neural mechanisms in crickets. In honey bees [40,41], cockroaches [42] and locusts [43,44], prominent NOS activity has been observed in neurons in the optic lobe (primary visual center), the central complex, the mushroom body (multisensory associative center) and the antennal lobe (primary olfactory center), as well as some other areas of the brain. Results of more histochemical, electrophysiological, optophysiological, and pharmacological studies on neurons in these brain areas should extend our knowledge of the molecular basis and neural basis of LTM formation in visual learning.
